# Effect of Mycobacterial Drug Resistance Patterns on Patients’ Survival: A Cohort Study in Thailand

**DOI:** 10.5539/gjhs.v5n6p60

**Published:** 2013-08-22

**Authors:** Amornrat Anuwatnonthakate, Sara J. Whitehead, Jay K. Varma, Udomsak Silachamroon, Yuthichai Kasetjaroen, Saiyud Moolphate, Pranom Limsomboon, Jiraphun Inyaphong, Narin Suriyon, Suporn Kavinum, Navarat Chiengson, Phatchara Tunteerapat, Jaranit Kaewkungwal

**Affiliations:** 1 Department of Tropical Hygiene, Faculty of Tropical Medicine, Mahidol University, Bangkok, Thailand; 2 Thailand MOPH – U.S. CDC Collaboration, Nonthaburi, Thailand; 3 Department of Health, New York, United States of America; 4 Department of Clinical Tropical Medicine, Faculty of Tropical Medicine, Mahidol University, Bangkok, Thailand; 5 Thailand Ministry of Public Health, Nonthaburi, Thailand; 6 Research Institute of Tuberculosis, RIT- JATA, Chiang Rai, Thailand; 7 Provincial Health Office, Phuket, Thailand; 8 Office of Disease Prevention and Control 7, Ubon Ratchathani, Thailand; 9 Provincial Health Office, Chiang Rai, Thailand; 10 Provincial Health Office, Tak, Thailand; 11 Department of Health, Bangkok Metropolitan Administrative Area, Bangkok, Thailand; 12 Infectious Disease Institute, Nonthaburi, Thailand

**Keywords:** tuberculosis, drug resistance, mortality, survival, Thailand

## Abstract

**Background:**

Drug resistance substantially increases tuberculosis (TB) mortality. This study aimed to describe the prevalence of mycobacterial drug resistance pattern and association of common resistance patterns with TB mortality in Thailand.

**Method:**

A retrospective cohort study was conducted using TB surveillance data. A total of 9,518 culture-confirmed, pulmonary TB patients registered from 1 October 2004 to 31 December 2008 from the Thailand TB Active Surveillance Network were included in this study. Patients were followed up until TB treatment completion or death. Mycobacterial drug resistance patterns were categorized as pan-susceptible, rifampicin resistance, isoniazid monoresistance, and ethambutol/streptomycin resistance. Drug susceptibility testing (DST) was determined by Mycobacterial Growth Indicator Tube (MGIT) liquid culture systems. Survival analysis was applied.

**Result:**

Isoniazid monoresistance was the most common pattern, while rifampicin resistance had the largest impact on mortality. Cox regression analysis showed a significantly higher risk of death among patients with rifampicin resistance (adjusted hazard ratio (aHR) 1.9, 95% confident interval (CI), 1.5-2.5) and isoniazid monoresistance (aHR 1.4, 95% CI 1.1-1.7) than those with pan-susceptible group after adjustment for age, nationality, human immunodeficiency virus (HIV) and antiretroviral therapy (ART) status, diabetes mellitus, cavitary disease on chest x-ray, treatment observation, and province. HIV co-infection was associated with higher mortality in patients both on ART (aHR 1.9, 95% CI 1.5-2.5) and not on ART (aHR 8.1, 95% CI 6.8-9.8).

**Conclusion:**

Rifampicin resistance and isoniazid monoresistance were associated with increased TB mortality. HIV-coinfection was associated with a higher risk of death including among those taking antiretroviral therapy.

## 1. Introduction

Drug-resistant tuberculosis (TB) has emerged as an important global public health threat. World Health Organization (WHO) estimated 490,000 multi-drug resistant TB (MDR-TB) cases occurring every year in 2002-2006, with more than 110,000 deaths, predominantly in Eastern Europe and Asia (WHO, 2008). Thailand was ranked 18^th^ on the WHO’s list of 22 “high-burden” TB countries (WHO, 2010). In Thailand, the overall national rate of MDR-TB is estimated to be 1.7% among new patients and 35% among retreatment cases respectively (Jittimanee et al., 2009; WHO, 2010). The national drug resistance surveys in 2002 and 2006 found the rate of MDR-TB among new TB patients had increased from 1.0% to 1.7%; resistance to at least isoniazid remained stable (9.5% and 9.7%), and resistance to rifampicin had increased from 1.4% to 2.6% (WHO South-East Asia Regional Office, 2008). Additionally, approximately 16% of Thailand’s TB cases are estimated to be HIV-associated.

The prevalence of MDR-TB in Thailand is increased especially in high HIV prevalence areas and in high risk populations such as prisoners and migrants compared to the non-HIV and non-prisoner population (Nishiura, Patanarapelert, & Tang, 2004; Pleumpanupat et al., 2003; Tansuphasiri, Pleumpanupat, Pandii, & Rienthong, 2003; TB Bureau, 2008). In Thailand, most studies have not confirmed HIV infection as a risk factor for MDR-TB (Akksilp et al., 2009; Maranetra, 1996; Tansuphasiri, Pleumpanupat, Pandii, & Rienthong, 2003; Boonsarngsuk, Tansirichaiya, & Kiatboonsri, 2009), while a study in northern Thailand found HIV infection was a risk factor for MDR-TB (Yoshiyama et al., 2001).

Globally, patients with MDR-TB have lower cure rates and higher mortality rates than patients with drug-susceptible TB (Mitnick et al., 2003; Nathanson et al., 2006). This is also true in Thailand, where MDR-TB has a significant association with mortality rate both in HIV-infected and HIV-uninfected TB patients (Maranetra, 1996; Amnuaiphon et al., 2009; Sanguanwongse et al., 2008).

In health care systems with effective TB care and control programs, the success rate for drug-susceptible TB treated with first-line anti-TB medications is 95%, but only 60-70% for MDR-TB or rifampicin resistant TB and 30% for extensively drug resistant TB (XDR-TB) (Blondal, 2007; Kliiman & Altraja, 2009; Orenstein et al., 2009). A study in Denmark showed that high- and low- level isoniazid resistance did not affect treatment outcome of standard treatment (Bang et al., 2010), and treatment outcomes for isoniazid monoresistant cases in United States were excellent and no different than for drug susceptible cases (Cattamanchi et al., 2009). A study in rural South Africa, by contrast, found poor outcomes among 16% of isoniazid monoresistant TB patients, 61% of who progressed to MDR-TB (Jacobson et al., 2011).

The Thailand Active TB Surveillance Network (TB-Net) was an initiative conducted from 2004-2011 to enhance surveillance, monitoring, evaluation and treatment of TB in 5 provinces in Thailand. This study aims to describe prevalence of drug resistance and risk factors for mortality in the TB-Net project area.

## 2. Methods

### 2.1 Setting

The TB-Net was a partnership between the Thailand Ministry of Public Health (MOPH), Bangkok Metropolitan Administration (BMA), U.S. Centers for Disease Control and Prevention (CDC), and Research Institute of Tuberculosis, Japan. The catchment area included nine districts in Bangkok and all districts in four provinces (Chiang Rai, Phuket, Ubon Ratchathani and Tak), and the national infectious diseases hospital in Thailand. The catchment area included 68 public and 27 private health care facilities. Five laboratories in TB-Net were supported to develop the capacity to perform liquid mycobacterial culture and identification, of which three (BMA, Ubon Ratchathani and the national TB program’s reference laboratory (NTRL)) have the capacity to conduct drug susceptibility testing.

### 2.2 Study Population

Patients with culture-confirmed, pulmonary TB patients enrolled in the TB-Net sites during the period 1 October 2004 to 31 December 2008 and with drug susceptibility test results available were included in this study. All TB cases were eligible for analysis if they were registered during the study period, were diagnosed with pulmonary TB (PTB), and were new or previously treated cases. We excluded those who transferred in from a different healthcare facility, had extrapulmonary TB or did not have sputum AFP smear, culture, or DST performed. All patients were followed up until the end of TB treatment or death. Drug-resistant TB was defined and categorized through laboratory confirmation of in vitro resistance to one or more first-line anti-tuberculosis drugs (WHO, 2008). Patients were classified according to TB registration type, previous TB history, and TB treatment outcome using standard WHO definitions (WHO, 2010).

### 2.3 Definitions

*Pan-susceptible:* TB strains susceptible to four first line anti-TB drugs including isoniazid, rifampicin, ethambutol and streptomycin.

*Multi-drug resistance* (MDR TB): TB strains resistant to at least isoniazid and rifampicin, with or without resistance to additional drugs.

*Rifampicin resistance*: TB strains resistant to rifampicin with or without other resistance, i.e. including MDR-TB, rifampicin monoresistance, and rifampicin with streptomycin and/or ethambutol resistance.

*Isoniazid monoresistance*: TB strains resistant to isoniazid, and susceptible to other first line anti-TB drugs.

*Ethambutol/streptomycin resistance*: TB strains resistant to ethambutol and/or streptomycin and susceptible to rifampicin.

*Death*: Having died for any reason during TB treatment including second line treatment of any duration.

*Survival*: Being alive at the end of TB treatment (outcomes classified as cured, completed, or failed). Patients with outcomes of “transferred out” and “defaulted” were excluded from survival analysis.

*Survival time*: duration of time from TB treatment initiation to date of death; calculated from date of death or end of TB treatment minus the date of TB treatment initiation. Times are censored at the end of TB treatment.

### 2.4 Drug Susceptibility Testing

For people diagnosed with TB, healthcare facilities were asked to submit at least one sputum specimen for culture in both smear-positive and smear-negative pulmonary TB patients, ideally during the first month of TB treatment; sputum culture was not routinely used to diagnose TB. All specimens were cultured on both Lowenstein–Jensen and Mycobacterial Growth Indicator Tube (MGIT, Becton-Dickinson), and positive cultures were identified using either biochemical tests in 2004 to 2007 or rapid immunochromatographic assay (Capillia TB) starting in 2008 (Ngamlert et al., 2009). Isolates identified as *Mycobacterium tuberculosis* (MTB) were sent for DST on MGIT using standard methods for first-line drugs (streptomycin, isoniazid, rifampin, ethambutol) at BMA central laboratory for TB patients in Bangkok, NTRL for province-level laboratories, or Ubon Rachathani regional disease control laboratory for TB patients in Ubon Ratchatani (Srisuwanvilai et al., 2008; Varma et al., 2007).

### 2.5 Data Collection

Network sites used standardized surveillance forms or modified TB registration forms to collect TB patients baseline demographic characteristics and clinical information, including symptoms, chest x-rays, HIV status and HIV treatment, initial TB treatment regimen, initial mycobacterial test results (smear, culture, identification, DST), treatment outcomes; these were entered into an electronic database (Varma et al., 2007).

### 2.6 Data Management and Analysis

We linked demographic and clinical data with laboratory data to verify culture and DST results. We resolved conflicting results by reviewing network site and NTRL source records. The NTRL’s results of culture and DST were used in the case of discordant records.

Thai TB patients who had the Thai national identification number were cross checked with the vital registration system of the Ministry of Interior to identify unreported deaths. TB patients with outcomes of “defaulted” and “transferred out” whose identification numbers were identified in the vital registration database at least 1 month after TB treatment initiation were classified with an outcome of “death” when the date of death was before or on the date of treatment outcome. There were 26 (2.9%) additional deaths identified through vital registration records compared to TB surveillance data.

A Kaplan-Meier survival analysis was applied to assess survival by drug resistance pattern. A Cox regression model was used to identify factors influencing survival time among TB patients with different drug resistance patterns. We chose variables for a Cox proportional hazards model bases on plausibility, previously published evidence, or a *P* value <0.2 in bivariate analysis. SPSS version 15.0 and Stata 10.0 (College Station, TX, USA) were used. Statistical significance for all comparisons were based on an alpha level <0.05 with 95% confidence interval.

### 2.7 Ethical Approval

This study was reviewed and approved by ethics committee of the Faculty of Tropical Medicine, Mahidol University number MUTM 2009-039-01 on September 25, 2009. The protocol for the Thailand TB Active Surveillance Network was reviewed by the Thailand Ministry of Health and U.S. CDC and found to be surveillance and public health program implementation, and not human subjects research requiring oversight by an institutional review board.

## 3. Results

### 3.1 Characteristics of TB Patients Stratified by Anti-TB Drug Resistant Patterns

Among 9,736 patients enrolling with TB in the TB Network sites during the study period, 9,518 had culture and DST information available for review; 8,790 were new cases and 728 previously treated. Of 8,790 new TB patients enrolling during the study period, 7,218 (82.1%) showed no resistance to first line TB drugs, while 240 (2.7%) were rifampicin resistant, 625 (7.1%) isoniazid monoresistant, and 707 (8.0%) ethambutol/streptomycin resistant. Of 728 patients who had been previously treated for TB, 466 (64.0%) showed no resistance to first line TB drugs, while 118 (16.2%) were rifampicin resistant, 66 (9.1%) isoniazid monoresistant, and 78 (10.7%) ethambutol/streptomycin resistant. Among 358 patients with rifampin resistance, 264 (73.7%) had MDR-TB, 70 (19.6%) rifampicin monoresistance, and 24 (6.7%) rifampicin resistance combined with either streptomycin or ethambutol resistance. Characteristics of TB patients are shown in Table 1, by drug resistance pattern.

**Table 1:** Characteristics of TB patients, stratified by drug susceptibility testing patterns, the Thailand TB Active Surveillance Network, Oct 2004- Dec 2008

Characteristics	Drug susceptibility testing patterns				Total No. (%) (N=9,518)

	Pan-susceptible No. (%) (N=7684)	Rifampicin resistance No. (%) (N=358)	Isoniazid monoresistance No. (%) (N=691)	Ethambutol/streptomycin resistance No. (%) (N=785)	
Type of TB registration					
New cases	7218 (93.9)	240 (67.0)	625 (90.4)	707 (90.1)	8790 (92.4)
Retreatment cases	466 (6.1)	118 (33.0)	66 (9.6)	78 (9.9)	728 (7.6)
Type of pulmonary TB					
Smear-positive	6860 (89.3)	319 (89.1)	638 (92.3)	718 (91.5)	8535 (89.7)
Smear-negative	824 (10.7)	39 (10.9)	53 (7.7)	67 (8.5)	983 (10.3)
Age (years)					
0-14	60 (0.8)	1 (0.3)	4 (0.6)	12 (1.5)	77 (0.8)
15-44	4043 (52.6)	230 (64.2)	344 (49.8)	456 (58.1)	5073 (53.3)
45-64	2386 (31.1)	91 (25.4)	224 (32.4)	212 (27.0)	2913 (30.6)
>65	1191 (15.5)	35 (9.8)	118 (17.1)	104 (13.2)	1448 (15.2)
Unknown/missing	4 (0.1)	1 (0.3)	1 (0.1)	1 (0.1)	7 (0.1)
Median age (years); (IQR^π^)	43 (32.0-56.0)	39 (29.0-50.0)	44 (34.0-57.3)	40 (29.3-54.0)	43 (31.0-56.0)
Sex					
Male	5244 (68.2)	229 (64.0)	503 (72.8)	562 (71.6)	6538 (68.7)
Female	2440 (31.8)	129 (36.0)	188 (27.2)	223 (28.4)	2980 (31.3)
Nationality					
Thai	6898 (89.8)	300 (83.8)	626 (90.6)	698 (88.9)	8522 (89.5)
Non-Thai	786 (10.2)	58 (16.2)	65 (9.4)	87 (11.1)	996 (10.5)
Marital status					
Married	4619 (60.1)	173 (48.3)	405 (58.6)	437 (55.7)	5634 (59.2)
Non-married	2870 (37.4)	173 (48.3)	264 (38.2)	335 (42.7)	3642 (38.3)
Unknown/missing	195 (2.5)	12 (3.4)	22 (3.2)	13 (1.7)	242 (2.5)
Mobile population^§^					
Non-mobile	6143 (79.9)	248 (69.3)	529 (76.6)	540 (68.8)	7460 (78.4)
Mobile	1296 (16.9)	98 (27.4)	143 (20.7)	224 (28.5)	1761 (18.5)
Unknown/missing	245 (3.2)	12 (3.4)	19 (2.7)	21 (2.7)	297 (3.1)
Had been treated with isoniazid preventive therapy					
Yes	117 (1.5)	31 (8.7)	16 (2.3)	21 (2.7)	185 (1.9)
No	7405 (96.4)	321 (89.7)	663 (95.9)	750 (95.5)	9139 (96.0)
Unknown/missing	162 (2.1)	6 (1.7)	12 (1.7)	14 (1.8)	194 (2.1)
Cough lasting > 2 weeks at time of diagnosis				
Yes	5532 (72.0)	263 (73.5)	512 (74.1)	540 (68.8)	6847 (71.9)
No	1961 (25.5.)	87 (24.3)	164 (23.7)	226 (28.8)	2438 (25.6)
Unknown/missing	191 (2.5)	8 (2.2)	15 (2.2)	19 (2.4)	233 (2.4)
Had used injection drugs					
Yes	185 (2.4)	15 (4.2)	15 (2.2)	21 (2.7)	236 (2.5)
No	7061 (91.9)	320 (89.4)	638 (92.3)	728 (92.7)	8747 (91.9)
Unknown/missing	438 (5.7)	23 (6.4)	38 (5.5)	36 (4.6)	535 (5.6)
Previously in prison					
Yes	205 (2.6)	16 (4.5)	24 (3.5)	23 (2.9)	268 (2.8)
No	7373 (96.0)	333 (93.0)	658 (95.2)	751 (95.7)	9115 (95.8)
Unknown/missing	106 (1.4)	9 (2.5)	9 (1.3)	11 (1.4)	135 (1.4)
Living in migrant labor camp					
Yes	278 (3.6)	9 (2.5)	24 (3.5)	25 (3.2)	336 (3.5)
No	7172 (93.3)	325 (90.8)	646 (93.5)	736 (93.8)	8879 (93.3)
Unknown/missing	234 (3.0)	24 (6.7)	21 (3.0)	24 93.1)	303 (3.2)
Diabetes mellitus					
Diabetes	487 (6.3)	25 (7.6)	47 (6.8)	55 (7.0)	614 (6.5)
No diabetes	678 (86.9)	306 (8.5)	604 (87.4)	691 (88.0)	8279 (87.0)
Unknown/missing	519 (6.8)	27 (7.5)	40 (5.8)	39 (5.0)	625 (6.5)
HIV and ART status					
Positive with ART	579 (7.5)	53 (14.8)	49 (7.1)	71 (9.0)	752 (7.9)
Positive without ART	717 (9.3)	57 (15.9)	73 (10.6)	84 (10.7)	931 (9.8)
Negative	5748 (74.8)	214 (59.8)	512 (74.1)	558 (71.1)	7032 (73.9)
Unknown/missing	640 (8.3)	34 (9.5)	57 (8.2)	72 (9.2)	803 (8.4)
Chest radiograph					
Normal	101 (1.3)	5 (1.4)	16 (2.3)	23 (2.9)	145 (1.5)
Cavity disease	2095 (27.3)	101 (28.2)	193 (27.9)	234 (29.8)	2623 (27.6)
Abnormal, no cavity	4884 (63.6)	227 (63.4)	436 (63.1)	468 (59.6)	6015 (63.2)
Missing/not performed	604 (7.9)	25 (7.0)	46 (6.7)	60 (7.6)	735 (7.7)
Initial treatment prescribed					
CAT I (2HRZE/4HR)	7197 (93.7)	250 (69.8)	642 (92.9)	723 (92.1)	8812 (92.6)
Other regimens	487 (6.3)	108 (30.2)	49 (7.1)	62 (7.9)	706 (7.4)
Treatment observer					
Healthcare worker	2164 (28.2)	90 (25.2)	176 (25.5)	210 (26.8)	2640 (27.7)
Family	4601 (59.9)	202 (56.4)	440 (63.7)	468 (59.6)	5711 (60.0)
Others/Self-administered	919 (12.0)	66 (18.4)	75 (10.9)	107 (13.6)	1167 (12.3)
Treatment outcome					
Cured	4713 (61.3)	117 (32.7)	405 (58.6)	464 (59.1)	5699 (59.9)
Completed	1202 (15.6)	48 (13.4)	73 (10.6)	133 (16.9)	1456 (15.3)
Failure	122 (1.6)	58 (16.2)	21 (3.0)	18 (2.3)	219 (2.3)
Died	690 (9.0)	65 (18.2)	87 (12.6)	71 (9.0)	913 (9.6)
Default	642 (8.4)	41 (11.5)	73 (10.6)	65 (8.3)	821 (8.6)
Transferred out	312 (4.1)	26 (7.3)	30 (4.3)	32 (4.1)	400 (4.2)
On treatment	3 (0)	3 (0.8)	2 (0.3)	2 (0.3)	10 (0.1)
Site					
BMA	1506 (19.6)	101 (28.2)	162 (23.4)	250 (31.8)	2019 (21.2)
Infectious Disease Institute	304 (4.0)	36 (10.1)	20 (2.9)	35 (4.5)	395 (4.2)
Ubon Ratchathani	2086 (27.1)	75 (20.9)	214 (31.0)	133 (16.9)	2508 (26.4)
Chiang Rai	2423 (31.5)	72 (20.1)	193 (27.9)	258 (32.9)	2946 (31.0)
Phuket	868 (11.3)	37 (10.3)	72 (10.4)	71 (9.0)	1048 (11.0)
Tak	497 (6.5)	37 (10.3)	30 (4.3)	38 (4.8)	602 (6.3)

§ Mobile was defined as not living in the same district for at least three of the past six months

π IQR= Inter Quartiles Range

H= isoniazid, R= Rifampicin, Z= Pyrazinamide, E= Ethambutol

Overall, patients with rifampicin resistance tended to be younger and a higher proportion were female compared to those with ethambutol/streptomycin resistance. The prevalence of HIV co-infection was 17.7% overall, and 30.7% among patients with rifampicin resistance, 17.7% among patients with isoniazid monoresistance, 19.7% among patients with ethambutol/streptomycin resistance, and 16.8% among patients with pan-susceptible TB. Retreatment cases accounted for 6.1%, 33.0%, 9.6%, 9.9% of pan-susceptible, rifampicin resistant, isoniazid resistant, ethambutol/streptomycin resistant, and pan-susceptible cases respectively. Overall the TB treatment success rate (combined cure and completion) was 66%. The success rate by drug resistance pattern was 76.9%, 46.1%, 69.2%, and 76.0% among pan-susceptible, rifampicin resistant, isoniazid resistant, ethambutol/streptomycin resistant, and pan-susceptible cases respectively.

### 3.2 Risk Factors for Mortality in Drug-Resistant TB Patients at the End of TB Treatment by Cox Proportional Hazard Model

In multivariate Cox proportional-hazards regression, several factors were significantly associated with mortality: older age, Thai nationality, absence of diabetes mellitus, HIV with or without ART, site, and drug resistance pattern (Table 2). Patients with either rifampicin resistance (aHR 1.9, 95% CI 1.5–2.5) or isoniazid monoresistance (aHR 1.4, 95% CI 1.1–1.7) had a higher risk of mortality compared with patients who had pan-susceptible TB.

**Table 2:** Risk factors for mortality among TB patients receiving drug susceptibility testing from treatment start to the end of TB treatment, the Thailand TB Active Surveillance Network, Oct 2004- Dec 2008

Factors	Died/Total n(%)	^¶^HR (95% CI‡)	Adjusted HR (95% CI)
Type of TB registration			
New cases	816/8790 (9.3)	Referent	NS
Retreatment cases	97/728 (13.3)	1.3 (1.0-1.6)	
Type of pulmonary TB			
Smear-positive	834/8535 (9.8)	Referent	NS
Smear-negative	7/83 (8.0)	0.8 (0.7-1.0)	
Age (years)			
15-44	338/5073 (6.7)	Referent	Referent
0-14	5/77 (6.5)	1.0 (0.4-2.4)	1.2 (0.5-2.)
45-64	272/2913 (9.3)	1.4 (1.2-1.7)	2.1 (1.8-2.5)*
≥65	298/1448 (20.6)	3.4 (2.-4.0)	5.6 (4.6-6.8)*
Sex			
Female	261/2980 (8.8)	Referent	NS
Male	652/6538 (10.0)	1.1 (1.0-1.3)	
Nationality			
Non-Thai	26/96 (2.6)	Referent	Referent
Thai	887/8522 (10.4)	3.7 (2.5-5.5)	3.4 (2.3-5.1)*
Marital status			
Married	492/5634 (8.7)	Referent	NS
Non-married	414/3642 (11.4)	1.3 (1.2-1.5)	
Unknown/missing	7/242 (2.9)	0.3 (0.2-0.7)	
Mobile population^§^			
Mobile	127/1761 (7.2)	Referent	NS
Non-mobile	768/7460 (10.3)	1.4 (1.2-1.8)	
Unknown/missing	18/297 (6.1)	0.8 (0.5-1.4)	
Had been treated with isoniazid preventive therapy		
Yes	19/185 (10.3)	Referent	NS
No	878/9139 (9.6)	0.9 (0.4-1.7)	
Unknown/missing	16/194 (8.2)	1.0 (0.6-1.6)	
Cough lasting > 2 weeks at time of diagnosis		
Yes	598/6847 (8.7)	Referent	NS
No	290/2438 (11.9)	1.3 (0.9-1.9)	
Unknown/missing	25/233 (10.7)	1.0 (0.6-1.6)	
Had used injection drugs			
No	847/8747 (9.7)	Referent	NS
Yes	35/236 (14.8)	0.6 (0.9-1.9)	
Unknown/missing	31/535 (5.8)	1.5 (1.2-1.6)	
Previously in prison			
No	857/9115 (9.4)	Referent	NS
Yes	37/268 (13.8)	1.5 (1.0-2.4)	
Unknown/missing	19/135 (14.1)	1.4 (1.0-2.0)	
Living in migrant labor camp			
In camp	9/336 (2.7)	Referent	NS
Not in camp	876/8879 (9.9)	3.4 (1.6-7.2)	
Unknown/missing	28/303 (9.2)	3.7 (1.-7.2)	
Diabetes mellitus			
Diabetes	43/614 (7.0)	Referent	Referent
No diabetes	822/8279 (9.9)	1.5 (1.1-2.0)	1.5 (1.1-2.0)*
Unknown/missing	28/625 (7.7)	1.1 (0.7-1.7)	1.1 (0.7-1.8)
HIV and ART status			
Negative	458/7032 (6.5)	Referent	Referent
Positive with ART	76/752 (10.1)	1.4 (1.1-1.7)	1.9 (1.5-2.5)*
Positive without ART	256/931 (27.5)	4.8 (4.1-5.5)	8.1 (6.8-9.8)*
Unknown/missing	123/803 (15.3)	2.6 (2.1-3.3)	2.3 (1.9-2.9)*
Chest radiograph			
Normal	7/145 (4.8)	Referent	Referent
Cavity disease	170/2623 (6.5)	1.4 (0.7-3.0)	1.7 (0.8-3.7)
Abnormal, no cavity	629/6015 (10.5)	2.3 (1.1-4.9)	2.1 (1.0-4.5)
Missing/not performed	107/735 (14.6)	3.4 (1.6-7.4)	2.8 (1.3-5.9)
Initial treatment prescribed			
CAT I (2HRZE/4HR)	832/8812 (9.4)	Referent	NS
Other regimens	81/706 (11.5)	1.1 (0.8-1.3)	
Treatment observer			
Others/Self-administered	73/1167 (6.3)	Referent	Referent
Healthcare worker	224/2640 (8.5)	1.3 (1.0-1.7)	1.3 (0.-1.7)
Family	616/5711 (10.8)	1.7 (1.4-2.2)	1.4 (1.1-1.8)
Site			
BMA	102/2019 (5.1)	Referent	Referent
Infectious Disease Institute	62/395 (15.7)	3.2 (2.3-4.3)	2.2 (1.6-3.1)*
Ubon Ratchathani	227/2508 (9.1)	1.9 (1.5-2.4)	1.6 (1.3-2.1)*
Chiang Rai	38/2946 (13.2)	3.0 (2.4-3.7)	2.9 (2.3-3.6)*
Phuket	93/1048 (8.9)	1.8 (1.3-2.4)	2.4 (1.7-3.2)*
Tak	40/602 (6.6)	1.4 (1.0-2.0)	2.6 (1.8-3.8)*
Drug susceptibility testing patterns			
Pan-susceptible	690/7684 (9.0)	Referent	Referent
Ethambutol/streptomycin resistance	71/785 (9.0)	0.9 (0.7-1.2)	1.0 (0.8-1.3)
Rifampicin resistance	65/358 (18.2)	1.8 (1.4-2.3)	1.9 (1.5-2.5)*
Isoniazid monoresistance	87/691 (12.6)	1.4 (1.1-1.7)	1.4 (1.1-1.7)*

¶ HR= Hazard ratio

‡ 95% CI= 95% confidence interval

* P-value < 0.05

§ Mobile was defined as not living in the same district for at least three of the past six months.

H= isoniazid, R= rifampicin, Z= pyrazinamide, E= ethambutol, S= streptomycin

### 3.3 Survival Time of TB Patients with Drug Susceptibility Testing after Initiation of TB Treatment

Among 9,518 TB patients with drug susceptibility testing, 7,138 (75%) survived for 888 days. The incidence rate of mortality among drug resistant TB patients was 0.5 per 1000 patients per day.

**Figure 1:**
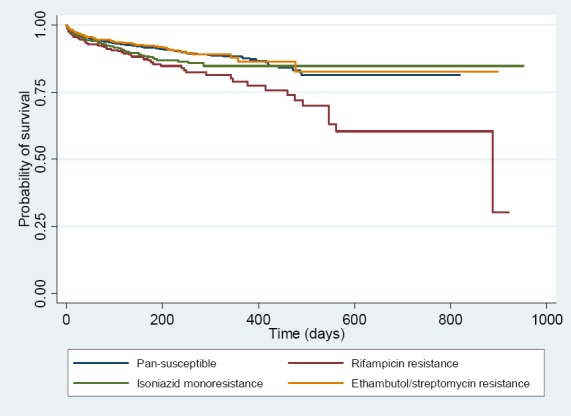
Survival rate of TB patients having drug susceptibility testing patterns including pan-susceptible, rifampicin resistance, isoniazid monoresistance, and ethambutol/streptomycin resistance

Kaplan-Meier survival estimates were calculated for the four drug resistance patterns. The survival rate differed significantly among four drug resistance patterns using a log-rank test (p-value <0.001) [Figure 1].

## 4. Discussion

Drug resistant tuberculosis is a global public health challenge, including in Thailand. While Thailand has a well-structured TB control program, poor treatment outcomes among TB patients with drug-resistant TB continue to be a problem (WHO, 2012). More information about the association between drug resistance patterns and mortality is needed to develop effective mechanisms to improve treatment outcomes in this population. This large cohort study of TB patients in Thailand sought to identify significant associations between drug resistance patterns, patient demographics and mortality. Our analyses indicate that significant differences in mortality exist by drug susceptibility patterns and certain patient characteristics. Such information may help to reduce mortality related to drug resistant TB.

This study was unique as it included a representative cohort of TB patients with culture-confirmed drug resistance and well defined resistance patterns. After adjusting for age, nationality, HIV status, site of TB treatment provided, association with mycobacterial tuberculosis drug resistance and mortality was documented over 4 years of observation at 68 public and 27 private hospitals throughout Thailand. The survival rate among four drug susceptibility testing pattern categories was significantly different, especially after 200 days of observations (p<0.001, Figure 1). Among TB patients with four drug susceptibility testing patterns, the mortality rate was highest in those with rifampicin resistance, which included patients with MDR-TB and rifampicin monoresistance. The rate of successful treatment outcome was also lowest in the rifampicin resistant group. The current findings are consistent with the finding reported in Tak province, Thailand, and also concurrent with existing literature on TB drug resistance and higher death rate (Amnuaiphon et al., 2009; Hemhongsa et al., 2008, WHO, 2010).

Isoniazid monoresistance was more common, 691/1834 (37.7%) of drug-resistant cases, than rifampicin resistance in this study and accounted for the largest number of deaths (87/223, 39%). The Cox regression model in this study showed a 1.4-fold higher death rate for isoniazid monoresistant TB compared to pan-susceptible after adjusting for age, nationality, and HIV status. A similar distribution of isoniazid monoresistance and impact on mortality has been reported by other recent studies in Asia. Isoniazid monoresistance was associated with a higher death rate in an observational cohort of 7433 TB patients during 2000 to 2006 in Singapore (Low et al., 2009). Similarly high isoniazid resistance and impact on mortality was also reported in Iran (Marjani et al., 2012).

Isoniazid monoresistance being treated as drug-sensitive TB may lead to emergence and progression to MDR TB (Pinto & Menzies, 2011; Yashodhara et al., 2010). The clinical treatment guidelines for isoniazid monoresistance is still empirical and yet to be evidence-based (Pinto & Menzies, 2011; Yoshiyama et al., 2001).

Among provinces included in the study, Chiang Rai had a high prevalence of isoniazid resistance and the highest mortality rate (aHR2.9, Table 2). This might be due to its high HIV prevalence (Chaiyasirinroje et al., 2012; Kantipong, Murakami, Moolphate, Aung, & Yamada, 2012).

Patient characteristics associated with increased mortality were age above 44 years and Thai nationality. The association of older age with mortality is well recognized and our finding was consistent with other studies in Thailand (Amnuaiphon et al., 2009; Moolphate et al., 2011). The association of Thai nationality is difficult to explain. The yield of vital registration linkage for Thai citizens only resulted in 26 (2.9%) additional deaths identified, so this does not account for the association. There may be unmeasured confounding by sociodemographic status or a healthy worker effect among migrant workers present in this cohort. Not having diabetes mellitus was another non-intuitive factor associated with death. It is possible that those with diabetes received more adherence support from healthcare staff. This association requires further investigation to explain.

In this study, the strong synergistic effect of HIV and drug resistance on mortality was observed in the Cox regression model: aHR1.9 in ART treated patients and aHR8.1 in non-ART treated patients. These findings further emphasize the important challenges of addressing HIV co-infection and drug resistance not only in Thailand but also in all the TB HIV endemic settings around the world (WHO, 2012; WHO, 2010).

## 5. Limitations

This study was conducted using secondary data from a surveillance project within the routine public health system in participating sites. The drug susceptibility tests were supported only for pulmonary TB patients and among this population, varied according to clinician practice. We collected the initial DST; follow up DST results and regimen changes were not available in the dataset. Despite good quality control practices in the five participating laboratories, there were some contaminated and missing culture results, which may result in misclassification. Vital registration data were not available among non-Thai citizens and among those who had lost their Thai citizen ID number or who lacked an ID number. Treatment outcomes in the TB register and surveillance data were used for such cases. Lastly, we included TB patients who were diagnosed with any form of drug resistant TB. However, the treatment protocol of the clinicians, different health service facilities, availability of second line drugs, alertness of the clinicians to drug resistance reports, and patients’ adherence to the therapy were variable and beyond the control of the investigators in this surveillance- based cohort evaluation.

## 6. Conclusion

Rifampicin and isoniazid resistance was associated with a significant increase in mortality among our population of TB patients. Increased mortality associated with isoniazid monoresistance raises concerns about empiric treatment recommendations in this group. In addition, certain previously reported patient-associated risk factors for TB mortality were noted, and in particular, HIV co-infection had a profound impact on mortality in our patients with TB drug resistance. Current study findings emphasize the need to develop multidimensional interventions to reduce TB mortality in Thailand.
